# Therapeutic abortion in Iran: an epidemiologic study of legal abortion in 2 years

**DOI:** 10.1186/s13104-020-05098-y

**Published:** 2020-05-27

**Authors:** Seyed Amirhosein Mahdavi, Asieh Jafari, Khadijeh Azimi, Nikoo Dehghanizadeh, Abdolrazagh Barzegar

**Affiliations:** 1 Legal Medicine Research Center, Iranian Legal Medicine Organization, Tehran, Iran; 2 Nursing and Midwifery Care Research Center, Tehran University of Medical Sciences, Tehran, Iran

**Keywords:** Legal abortion, Fetal anomaly, Maternal disease

## Abstract

**Objectives:**

Unsafe abortion is one of the most important causes of death and disability among mothers in countries where abortion is illegal. These conditions have changed since then. The present study has investigated the cases who were referred to the legal medicine organization to receive abortion permission. This country level secondary patient data analysis, investigated all the cases who were referred to the legal medicine centers of Iran for abortion permission during 2015 to 2017.

**Results:**

From 21,477 applicants, 15,617 (72.71%) received permission including 14,367 (91.99%) for fetal abnormalities and 1250 (8.01%) for maternal diseases. The most common fetal abnormalities/disorders were nervous system malformations (26.4%), chromosomal abnormalities (18.4%) and of maternal diseases were circulatory system diseases (43.9%), neoplasms (13.4%) and genitourinary system diseases (9.9%). The most common reasons for not permission were lack of supplementary documents to prove (38.8%), not competency with the criteria (33.9%), and gestational age of more than 19 weeks (25.8%).

## Introduction

Annually, about 140 million pregnancies would occur out of which about 25% would be terminated before the fetus reaches viability due to spontaneous or induced abortion [1]. Induced abortion would be performed due to various reasons such as having unwanted pregnancy, mother’s or father’s unwillingness to have a child, diagnosing a fetal anomaly or disorder or a maternal disease [2, 3] and it is known as therapeutic abortion when there is a medical need for terminating the pregnancy (before 20th weeks) to save the mother’s life, prevent any mental or physical harm to the mother, or in cases where the fetus is diagnosed with a disorder or disease that would make it nonviable, or to reduce the number of fetuses in multifetal pregnancies [2].

Although the exact data about abortion in Iran is not available due to the inherent difficulty in reaching all diverse populations of Iran and the sensitivity of the issue, some researchers have tried to estimate the rate of various types of abortion in Iran. Erfani [4], showed that between 2009 and 2014, the proportion of having lifetime abortion in married women, the general abortion rate, the total abortion rate and the annual number of abortions in married women aged 15–49 who completed the 2009 Tehran Survey of Fertility decreased, respectively, from 8.3 to 5.6%, from 6.6 to 5.4 abortions per 1000 women, from 0.18 to 0.17 abortions per married woman and from 10,656 to 8734. However, the proportion of terminations obtained for nonmedical reasons rose from 68 to 81% [4]. In a 2012 meta-analysis by Motaghi et al. abortion was estimated at 8.9 per 1000 women aged 15 to 44 years and 5.34 per 100 live births [5]. According to a study by Rastegar et al. [6] in Iran in 2012, abortion rates with and without medical reasons were estimated to be 70.5 and 116.9 per 1000 pregnancies, respectively. In a 2015 study by Hosseini et al. women aged 15 to 49 years reported a 3.8% rate of induced abortion [7]. Ghofrani et al. [8] estimated the prevalence of induced miscarriage by both randomized response technique and unmatched count technique 14% and 12%, respectively.

In countries where induced abortion is illegal and there are very restrictive rules about abortion, women would seek unsafe abortion [9–12] which is one of the main causes of morbidity and death among the women around the world and is the reason behind the death of 8–18% of pregnant mothers and one eighth of the occurred complications in pregnancy [13] as well as psychological, socio political and judicial consequences [14]. According to a study by Majlessi et al. [15], 12% of 417 referrals for abortion in a hospital in Isfahan declared illegal abortion. So, we can come to the conclusion that one of the ways to prevent unsafe abortion is to enact the required laws that would allow women to have access to healthy abortion in the necessary cases [16, 17]. Induced abortion, at all ages, before or after viability [18], and in the entire world, has always encountered ethical, philosophical, biological, religious and legal challenges [16, 19]. Currently, the laws related to abortion are different in various countries and cover a range from full ban to full freedom [20, 21].

In Iran, for the first time in 1997 the permission for abortion was issued only for fetal major thalassemia and anencephaly cases before ensoulment which was estimated to be around 19 weeks of pregnancy. In 2002, the national committee of abortion approved a regulation with limited number of definitive maternal and fetal indications suggested for therapeutic abortion. According to the Single Article Act of therapeutic abortion (June 2005), wide range conditions for receiving permission for abortion and relating processes have been defined. According to this Act, therapeutic abortion is legally authorized with the consent of the pregnant mother after definite diagnosis of fetal anomaly or a life threatening maternal disease, by three medical specialists and its confirmation by the legal medicine organization before 19 weeks of pregnancy [22, 23].

According to the study by [24], abortion permission was issued for 51.4% of the 245 cases [24]. Following the evolution of regulations in 2002, Sadr et al. [25] surveyed all abortion applicants referring to legal medicine centers in the country in 2004 reporting that out of 1101 permission issued, 35.8% were for maternal and 63.9% for fetal cases. Since the last amendment to the law in 2005, dispersed provincial studies have been conducted. Sharifi et al. [26] surveyed all 428 abortion applicants in Kermanshah between 2005 and 2010, reporting 82.7% of permits, including 81% fetal and 19% maternal. According to a 6 years study of 1664 cases of abortion applicants in Fars Province, 79.6% were fetal and 20.4% maternal [27, 28]. According to the study by Vasegh et al. [29], for 48% of the 1378 female applicants for abortion referred to the legal medicine department of Tehran province, permissions were issued during a 1 year period 2011–12, of which 90.2% were fetal and the rest were maternal. In the Isfahan province study, over a 4 year period from 2011 to 2014, 629 of 830 (75.8%) referred women for fetal causes has been received the abortion permission [30]. In Hormozgan province, abortion was permitted in 77.9% of the 281 clients in a 1 year period of 2016–17 [31]. Studies show that the number of abortion applicants and the percentage of licensing has increased over the time.

Currently, if in the primary screenings and investigations by the prenatal caregivers (gynecologists and midwives) any fetal or maternal problem would be detected, the pregnant mother would be referred to the selected special center of legal medicine in the province and after receiving the permission, she would be referred to the hospital with the permission to have the safe abortion under the supervision of healthcare providers. So, it is more than two decades that therapeutic abortion is being performed in Iran safely and legally and it has created a ground for decreasing the number of unsafe abortions and their complications and also decreasing the burden of diseases that continuing pregnancy would cause.

Considering that no comprehensive country level study has been conducted after 2006, while abortion laws in the country have changed significantly. The present study has been conducted to investigate all requests for legal abortion throughout the country by detail including the frequency of issued permissions and the frequency of fetal and maternal causes.

## Main text

### Methods

The present study is a secondary patient data analysis in Iran. In this study data of all the clients who were referred to centers of legal medicine in all 31 provinces in the country to receive permission for abortion from March 21st, 2015 to March 20th, 2017 (2 years) have been used as the study data.

Data was gathered using a researcher-made checklist. To develop the checklist, first the researcher evaluated the files of 20 cases who had requested permission for abortion and extracted the maximum accessible information based on which the checklist for the study was developed. At the next stage, the checklist was evaluated by 8 experts from legal medicine organization and its validity was confirmed. In order to investigate its reliability, the information of 20 related files were extracted and evaluated again by 2 separate researchers and the agreement rate of 98% was achieved. One of the main variables was the cause of request including maternal, fetal and non-fetal/non-maternal. Cases that could not be put in the category of fetal or maternal anomalies/diseases were categorized as “non-fetal/non-maternal” which included cases such as drug consumption by the mother during pregnancy, being exposed to radiation during pregnancy, unwanted pregnancy and poor economic conditions. Considering the wide spectrum of fetal anomalies and also maternal diseases, they were categorized based on the classification of the diseases in ICD10 [32].

#### Data analysis

The Data were analyzed using SPSS 21 software and descriptive statistics included absolute and relative frequency, mean and standard deviation.

#### Ethical considerations

Ethical approval was obtained from the Ethics Committee of Iranian Legal Medicine Organization.

### Results

During 2 years, 21,477 pregnant mothers had been referred to the 31 legal medicine centers of Iran out of which, 83.6% were due to fetal, 12.7% due to maternal and 3.7% due to non-fetal/non-maternal causes. As shown in Table 1, 15,617 cases (72.71%) received permission for abortion out of which 14,367 cases (91.99%) were due to fetal causes and 1250 cases (8.01%) were due to maternal causes. None of the non-fetal/non-maternal cases received permission for abortion. Among the issued permissions for fetal causes, the most common anomalies were nervous system malformations (26.4%), chromosomal abnormalities (18.4%), hydrops fetalis (13.7%) and musculoskeletal anomalies (9.9%) respectively and in terms of maternal diseases, circulatory system diseases (43.9%), neoplasms (13.4%) and genitourinary system diseases (9.9%) were the most common diseases. The most common reasons that some pregnant women could not receive permission are shown in Table 2.

**Table 1:** Frequency of reasons for referral and final results during 2 years

Reasons for referral	Permitted N (%)	Not Permitted N (%)	Total N (%)
Fetal	14,367 (80)	3587 (20)	17,954 (83.6)
Maternal	1250 (45.9)	1474 (54.1)	2724 (12.7)
Non-fetal\non-maternal	0	799 (100)	799 (3.7)
Total	15,617 (72.71)	5860 (22.09)	21,477 (100)

**Table 2:** Frequency of reasons for not permitted cases

Reasons	Fetal N (%)	Maternal N (%)	Non-fetal\non-maternal N (%)	Total N (%)
Lack of enough supplementary documents to prove fetal anomaly/maternal disease	722 (20.2)	849 (57.6)	702 (87.8)	2275 (38.8)
No compliance with defined criteria	1354 (37.8)	569 (38.6)	64 (8)	1987 (33.9)
GA above 19 weeks	1457 (40.7)	43 (2.9)	9 (1.1)	1509 (25.8)
Intrauterine fetal death	47 (1.2)	5 (0.3)	8 (1.1)	60 (1)
Not approved pregnancy	7 (0.1)	8 (0.6)	16 (2)	31 (0.5)
Total	3579 (100)	1474 (100)	799 (100)	5860 (100)

The mean age of the mothers, among the clients, was about 30 in those who were referred due to fetal causes and was about 33 in cases due to maternal causes. Also 25% of the applicants for abortion were mothers older than 35 years. The mean gestational age was about 16 weeks in fetal cases and about 9 weeks in maternal cases (Table 3). Pregnancy in 21,159 cases (98.5%) was singleton. The number of twin pregnancies was 295 cases (1.4%) and the number of pregnancies higher than twins was 23 (0.1%). Also for 47 out of 48 conjoined twins’ permissions for abortion were issued. More than one third of the cases received the permission in the same day of referral and about one third of the cases received the permission in the next day. The mean and standard deviation mother age and gestational age was 28.76 ± 9.4 years and 15.7 ± 2.9 weeks in the case of permission and 29.18 ± 9.9 years and 14.9 ± 6.9 weeks in the case of non-permitted, respectively.

**Table 3:** Frequency of general characteristics of cases

Variable	Fetal N (%)	Maternal N (%)	Non-fetal\non-maternal N (%)	Total N (%)
Mother’s age (year)				
≤ 15	47 (0.3)	3 (0.1)	6 (0.8)	56 (0.3)
16–20	1182 (6.6)	57 (2.1)	28 (3.5)	1267 (5.9)
21–35	12,610 (70.2)	1598 (58.6)	590 (73.9)	14,798 (68.9)
36–45	4043 (22.5)	1043 (38.3)	172 (21.4)	5258 (24.5)
> 45	72 (0.4)	23 (0.8)	3 (0.4)	98 (0.5)
Total	17,954 (100)	2724 (100)	799 (100)	21,477 (100)
Mean ± SD	30.09 ± 6.56	33.48 ± 5.99	30.6 ± 6.18	30.59 ± 6.56
Gestational age (week)				
≤ 5	37 (0.2)	358 (13.1)	169 (21)	564 (2.5)
6–10	160 (0.9)	1546 (57)	455 (56.9)	2161 (9.8)
11–15	5852 (32.6)	531 (19.3)	97 (11.9)	6480 (30.2)
16–19	10,139 (56.5)	209 (7.6)	52 (6.6)	10,400 (48.7)
> 19	1766 (9.9)	80 (3)	26 (3.5)	1872 (8.8)
Total	17,954 (100)	2724 (100)	799 (100)	21,477 (100)
Mean ± SD	16.18 ± 2.27	9.79 ± 3.61	8.6 ± 4.4	15.48 ± 4.38

## Discussion

Generally, among the seven causes that could lead to issuing the permission for abortion around the world [21, 33], two reasons including preserving mother’s life and some of the fetal anomalies/diseases, lead to permission in Iran. The basis for issuing the permission for abortion in cases of fetal anomalies/diseases is lack of viability after birth or putting the mother’s life in too much trouble due to the fetal anomaly or the disease. In cases of maternal disease the basis for such permissions is the risk of the mother’s death if pregnancy continues [34]. The results of this study showed that three quarters (72.71%) of admitted cases could get the permission for abortion most of which have been due to fetal anomalies/diseases.

Also about half of the clients with maternal and four-fifths of the clients with fetal causes received permission for abortion. In some studies that have been done before the last revision of abortion law in Iran, the rate of proportion of permission were lower [24, 25, 35], but the results of subsequent studies were concordant with our findings [22, 27–29, 36]. In addition, a study that was conducted in a hospital in Turkey demonstrated that permission for abortion was issued for 63% of cases with fetal causes [37].

Two-thirds of fetal causes that led to issuing the permission were structural anomalies and one-third of them were genetic disorders. The prevalence of the anomalies in the studies that have been conducted in different provinces of Iran was also similar to the results of the present study [22, 28, 29, 38, 39]. Also, in the study of Kose et al. [37], the most common anomalies were nervous system anomalies, chromosomal disorders, cardiovascular and skeletal disorders and hydrops. Considering the desirable coverage of prenatal cares in Iran and also taking into account that in the health system of Iran, abortion without permission is illegal, after diagnosing any kind of fetal anomaly/disease, even minor ones, most of the cases would be referred to the legal medicine centers. Therefore, although there is no accurate statistics for the frequency of fetal anomalies in Iran, the estimated frequency could be considered very close to the real number of the fetal anomalies in Iran.

The requests of 27.29% of cases were rejected, out of which 25.8% were cases with major anomaly but gestational age of more than 19 weeks. It means that about 7% of all abortion requests were rejected because of gestational age of more than 19 weeks while it could be prevented. Mothers who do not receive the permission for abortion for a fetus with significant anomalies just because it is older than 19 weeks could be at risk of performing unsafe abortion [40]. Lack of information about the rules and regulations concerning abortion among the prenatal caregivers such as gynecologists, midwives, and general practitioners [41, 42] and also the society [43, 44] could be considered as reasons for delayed referral and therefore, their education in this matter should be taken into account.

Most cases with lack of competency with the criteria for fetal abortion were cases with minor anomalies. Many of fetal anomalies could be treated and corrected after birth. So, it is necessary that in case of facing with minor anomalies, mothers would be referred to perinatologists or neonatal experts so that the appropriate measures would be conducted in time.

Considering the Census of 2016 the number of women of reproductive age (15–50 years) in Iran was about 23 million and according to Erfani [4] the general abortion rate in 2014 was 5.4 abortions per 1000 women of reproductive age [4] so the number of abortions in 2016 could be calculated as 124,000 cases and considering the annual number of legal abortion license, 6.3% of which were legal and were consequently safe.

As a conclusion, during the 2-year period of the study, most of the client had referred due to fetal causes. Permission for abortion in most cases was issued due to fetal causes and in about half of the cases was issued for maternal causes. Also, for about one-quarter of the clients’ permission was not issued and one of the most important reasons for it was referring with the gestational age of above 19 weeks; which could totally be prevented. It is recommended that sufficient educations would be provided to gynecologists, midwives, and healthcare providers, who are responsible for taking care of the health of the pregnant mothers, so that they would consider this limited time window in scheduling the screening and tests.

## Limitation

As we used registered data, we were not able to access more in detail data including demographic data. Also, we could not compare our finding with the findings of other countries because of dissimilarity in therapeutic legislations.

## Data Availability

Data and material are available by contacting to corresponding author. In order to access the raw data, it is necessary to obtain the permission of Iranian Legal Medicine Directory.
